# Physical Activity Changes and Its Risk Factors among Community-Dwelling Japanese Older Adults during the COVID-19 Epidemic: Associations with Subjective Well-Being and Health-Related Quality of Life

**DOI:** 10.3390/ijerph17186591

**Published:** 2020-09-10

**Authors:** Yuta Suzuki, Noriaki Maeda, Daigo Hirado, Taizan Shirakawa, Yukio Urabe

**Affiliations:** 1 Department of Sports Rehabilitation, Graduate School of Biomedical and Health Sciences, Hiroshima University, Hiroshima 734-8553, Japan; yt.suzuki28@gmail.com (Y.S.); norimmi@hiroshima-u.ac.jp (N.M.); 2 Department of Rehabilitation, Matterhorn Rehabilitation Hospital, Hiroshima 737-0046, Japan; daigo0417tennis@gmail.com; 3 Department of Orthopedics, Matterhorn Rehabilitation Hospital, Hiroshima 737-0046, Japan; matter@jasmine.ocn.ne.jp

**Keywords:** coronavirus disease (COVID-19), lifestyle restrictions, elderly, physical activity, well-being, health-related quality of life

## Abstract

Psychological distress caused by decreased physical activity (PA) is a growing concern among the elderly due to public health measures since the coronavirus disease (COVID-19). We aimed to (1) assess how public health restrictions impact PA, subjective well-being (SWB), and health-related quality of life (HRQoL) of community-dwelling elderly, and (2) investigate risk factors that lead to a decline in PA. Self-administered questionnaires assessed the changes in PA, SWB, HRQoL. Multivariate logistic regression analysis was performed to identify significant associated risk factors for decreased PA. Of 165 participants (valid response rate, 41.3%; mean age, 78.5 ± 8.0 years), 47.3% became less active, 23.0% became more active, and 29.7% maintained PA levels. There was a significant decrease in SWB at baseline and follow-up after COVID-19 restrictions in the less active group (*p* < 0.01). Higher levels of moderate or strenuous exercise/sports activity at baseline (odds ratio [OR], 1.12; 95% confidence interval [CI], 1.01–1.24), and lower mental component HRQoL scores at baseline (OR, 0.96; 95% CI, 0.93–0.99) were associated with an increased risk of decreased PA. Public health restrictions impact the PA of the elderly, especially those who had higher levels of exercise/sports activity and lower HRQoL before COVID-19. Decreased PA was strongly associated with lower SWB.

## 1. Introduction

The coronavirus disease (COVID-19) outbreak was first reported in December 2019 [1], and has since spread to many countries around the world. On 11 March 2020, the World Health Organization (WHO) declared it a global pandemic, and on 16 April 2020, the Japanese government declared a nationwide state of emergency. The Japanese government closed schools and recreational and commercial facilities, and demanded that Japanese citizens stay at home as much as possible [2]. Social distancing was implemented nation-wide, requiring people to maintain a distance of 2 m between each other and avoid crowded places and non-essential social gatherings. These public health measures are expected to cause psychological problems. Studies have reported that people’s physical activity (PA) decreased during COVID-19 lockdown, and was associated with a decline in psychological health [3,4,5]; however, few studies focused on the elderly.

PA is defined as bodily movements produced by skeletal muscles that result in energy expenditure [6]. PA is important for elderly people, especially to maintain their level of independence, physical and mental health, and well-being [7,8]. Several studies have shown that PA has the potential to prevent symptoms of psychological health disorders such as depression and anxiety [9,10]. People who engage in more PA have a better health-related quality of life (HRQoL) [11]. Therefore, it is important for the elderly to maintain a healthy psychological state and HRQoL through maintenance of PA. However, it has not been investigated which elderly individuals are at greater risk of reduced PA, and which categories of PA will be affected due to COVID-19 restrictions. For example, elderly individuals who were originally engaged in more housework compared to outside exercise may maintain PA levels and, thus, keep good mental health.

To understand the factors that influence involvement in PA, it is necessary to consider personal, social, and environmental variables. Personal factors include internal motives such as exercise enjoyment and achievement, and are associated with exercise maintenance and improved sense of well-being [12]. Social factors include emotional and practical support from family, and environmental factors include availability and access to PA opportunities [13,14].

A detailed exploration of changes in PA behaviors in the elderly and an identification of the factors associated with a reduction in PA during the COVID-19 pandemic may inform public health policies promoting well-being and HRQoL in scenarios of public health restrictions. The purpose of this study is to understand the impact of public health restrictions on community-dwelling older adults since the onset of COVID-19, in relation to the changes in PA, subjective well-being (SWB), and HRQoL. Furthermore, this study aimed to investigate the personal, social, and physical environmental factors linked to a decline in PA. We hypothesized that the COVID-19 outbreak would have a negative impact on PA, SWB and HRQoL [3,5,11], and that personal and psychological factors, as well as social factors, would have affected PA levels.

## 2. Materials and Methods

### 2.1. Study Design and Setting

This study was conducted as a survey applied before and during the COVID-19 epidemic in Japan. Participants were extracted from the patient database of a convalescent rehabilitation hospital in Kure city, Hiroshima Prefecture.

### 2.2. Participants and Survey Procedures

We randomly selected 400 patients who met the following inclusion criteria from the patient database of the hospital: discharged from the convalescent rehabilitation hospital between January 2017 and December 2019 (a period of 3 years), or those who were visiting the outpatient department of the hospital during the same period, living independently at home at the time of the survey, and over 65 years old at the time of the survey.

This survey was conducted by mailing two types of self-administered questionnaires to their homes after the state of emergency was declared on 16 April 2020. The first questionnaire retrospectively assessed PA, SWB, and HRQoL in the four weeks before the declaration of the state of emergency for the baseline assessment (from 20 March–15 April), and the second was in the four weeks after the declaration of the state of emergency for follow-up (from 16 April–13 May). The first questionnaire included a survey of social demographics and baseline characteristics of the participants. Participants were requested to fill out both questionnaires and return them by 29 May. All participants signed an informed consent form approved by the Matterhorn Rehabilitation Hospital Institutional Review Board (approval number: MRH20002).

### 2.3. Measurement

#### 2.3.1. Social Demographics and Baseline Characteristics

Social demographics included age, gender, health history, occupational and hobby status, and family structure. Information on participants’ functional health, and the neighborhood was also obtained. 

The health history section investigated hypertension, diabetes, cardiovascular disease, cerebrovascular disease, and orthopedic disease. Participants selected one answer from “No”, “Regularly visiting the hospital or clinic”, “Cured” or “Neglected” in response to the question “Have you ever been diagnosed or treated for a particular disease?” When the respondents answered, “Regularly visiting the clinic” or “Neglected” they were classified as “Present”.

Regarding regular work and hobby activities before the declaration of emergency, participants selected “Yes” or “No”.

Family structure could be categorized as one of three types: “living alone”, “living only elderly couple” and “living with family”.

Functional health was assessed using the Tokyo Metropolitan Institute of Gerontology Index of Competence (TMIG-IC) [15]. The responses to each question were set as “Yes” (able to do) or “No” (unable to do), with a score of 1 for “Yes” and 0 for “No” The total score was the sum of 13 items, which indicated functional abilities of daily activities such as instrumental activities (e.g., shopping, preparing food, etc.), cognitive tasks (e.g., reading newspapers), and social activities (e.g., visiting friends). Functional health was classified as independent (13 points) or dependent (≤12 points) [16].

The neighborhood of the participants was evaluated using the Japanese version of the International Physical Activity Questionnaire Environment Module (IPAQ-E). In this study, we used three of the 17 questions that have been shown to be associated with PA in Japanese adults, including residential density, access to shops, and the presence of sidewalks [14]. The “neighborhood” was defined as an environment where a person could walk a 10–15 min radius of their home. The residential density was evaluated by the main type of housing in their neighborhood, and participants selected one of the following responses: “Detached single-family residences”, “Apartments, or condos of 2–3 story’s”, “Mix of single-family residences and apartments or condos”, “Apartments or condos of 4–12 story’s”, “Apartments or condos of more than 12 story’s”. The access to shops and the presence of sidewalks were evaluated by the following question: “Are most shops, stores, and markets for buying what you need within walking distance of your home?” and “Are there sidewalks on most of the streets in your neighborhood?” Respondents selected the answer from the following four response options: “Strongly disagree”, “Somewhat disagree”, “Somewhat agree” and “Strongly agree”. For the analysis, environmental variables were converted into dichotomous variables. For residential density, “Detached single-family residences” was categorized as low residential density, while the other selections were categorized as high residential density. The access to shops and the presence of sidewalks items were recategorized into agree (strongly agree and somewhat agree) and disagree (somewhat disagree and strongly disagree).

#### 2.3.2. Physical Activity Behavior

PA was assessed using the Physical Activity Questionnaire for Elderly Japanese (PAQ-EJ). The PAQ-EJ score was converted into a metabolic coefficient corresponding to task (MET) hours per week (MET h/week) in each of the seven subscales of transportation, light exercise/sports, moderate or strenuous exercise/sports, resistance exercise/sports, light housework, moderate or heavy housework, and labor [17].

In addition, in order to assess whether PA changed as a result of the COVID-19 pandemic, the following question was added in the second questionnaire: “Has your PA changed after the declaration of emergency?” Respondents selected from the three options: “Increased”, “About the same”, and “Decreased”, and responses were categorized as “More active”, “Equally active”, and “Less active”, respectively.

#### 2.3.3. Subjective Well-Being

SWB was assessed using the Japanese version of the World Health Organization’s (WHO) Five Well-being Index (WHO-5-J). The WHO-5-J measured the mental health of respondents in the previous two weeks and consisted of the following five items: (1) felt cheerful and in good spirits, (2) felt calm and relaxed, (3) felt active and vigorous, (4) woke up feeling fresh and rested, and (5) daily life filled with things that interest me [18]. The answers to each item were evaluated on a six-point Likert scale ranging from 0 to 5, with a maximum score of 25 points. Higher scores indicated better SWB.

#### 2.3.4. Health-Related Quality of Life

HRQoL was assessed using the Japanese version of the Medical Outcome Study 12-Item Short-Form Survey v2 (SF-12v2) questionnaire. The SF-12v2 is a well-known, globally used questionnaire with high reliability and validity for the measurement of HRQoL [19]. The SF-12v2 consists of 12 questions related to 8 different domains of quality of life: physical functioning, role limitations due to physical illness, bodily pain, general health perceptions, vitality, social functioning, role limitations due to emotional problems, and mental health. The score in each domain of the SF-12v2 was transformed into a numerical score ranging from 0 to 100, and calculated separately by the algorithm for the physical component summary score (PCS) and the mental component summary scores (MCS), with a higher score reflecting better self-perceived health.

### 2.4. Statistical Analysis

To analyze the changes in PA, SWB, and HRQoL associated with restrictions following the COVID-19 pandemic, two-way repeated-measures ANOVA with group (more, equally, less active) as a between-participants factor and with time (baseline and follow-up) as a within-participant factor were run. When interaction effects were detected, post-hoc comparisons were performed using a paired t-test to determine the differences in changes of variables before and during the COVID-19 epidemic in each group. We also compared variables before the COVID-19 epidemic among groups to compare the baseline.

In addition, we investigated the risk factors related to decreased PA due to the COVID-19 restrictions. Univariate analysis for each variable was conducted and the objective variable was “more/equally active” or “less active” classified based on self-reported changes in PA behavior. The variables that showed significant differences (*p* < 0.05) in univariate analysis were included as explanatory variables in the subsequent multivariate analysis. Multivariate analysis was performed using logistic regression to identify significant risk factors for decreased PA. To predict changes in PA, the variables of PA, SWB, and HRQoL at baseline were used for analysis. The level of significance for all analyses was set at *p* < 0.05. All data were analyzed using SPSS version 26.0 for Mac (SPSS Inc., Chicago, IL, USA).

## 3. Results

### 3.1. Selection of Participants

In all, there were 232 respondents (58.0% of all individuals contacted). Of these, 30 were notified that they could not respond through relatives, including 15 who had already died and 15 who were in nursing homes or care facilities. In addition, 12 respondents answered only one of two questionnaires, and 25 had incomplete or insufficient responses. This resulted in a final sample of 165 participants (valid response rate, 41.3%) that were included in the analysis.

### 3.2. Social Demographics and Baseline Characteristics

The social demographics and baseline characteristics of the participants are presented in Table 1. The mean age of all participants was 78.6 ± 8.0 years, and 69.7% (*n* = 115) were women. Half (*n* = 84, 50.9%) reported the presence of hypertension, and almost three-quarters (*n* = 76, 78.0%) reported the presence of orthopedic diseases. The majority of participants (*n* = 156, 94.5%) reported the absence of regular employment. The job descriptions for the nine elderly participants who engaged in regular work included five administrative assistants, one store employee, one cleaner, one security guard, and one fishery employee (data not shown). About half (*n* = 78, 47.3%) participated in hobby activities. The breakdown of hobbies included participation in local opportunities (*n* = 21; e.g., exercise classes or karaoke classes), gardening (*n* = 20), sports activities (*n* = 11; e.g., gateball, softball, table tennis, swimming, dancing or bowling), walking (*n* = 7), handicrafts (*n* = 6), language learning (*n* = 5), calligraphy (*n* = 3), reading books (*n* = 3), and fishing (*n* = 2) (data not shown). Approximately less than half (*n* = 72, 43.6%) lived as the elderly couple, and the other half lived alone or with their families, and about two-thirds were dependent on functional health (*n* = 96, 58.2%). In total, 86.7% (*n* = 143) lived in single-family residences (the data were presented as low residential density). Just over half (*n* = 85, 51.5%) lived in locations with convenient access to shops, and almost two-thirds (*n* = 101, 61.2%) reported the presence of sidewalks in their neighborhood.

### 3.3. Changes in Physical Activity before and during COVID-19 Restrictions

Based on self-reported changes in PA behavior, 47.3% (*n* = 78) of participants became less active while only 23.0% (*n* = 38) became more active, and 29.7% (*n* = 49) maintained their typical PA level.

**Figure 1:**
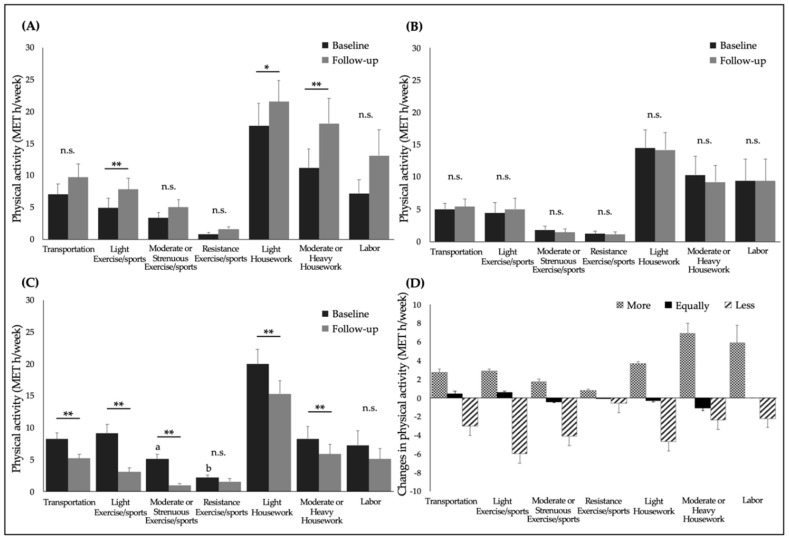
The changes in physical activity at baseline and follow-up after coronavirus disease (COVID-19) restrictions. (**A**) More active group (*n* = 38); (**B**) equally active group (*n* = 48); (**C**) less active group (*n* = 78); (**D**) changes in physical activity. Each category of physical activity was calculated in MET h/week. Data are expressed as the mean ± SEM. **: *p* < 0.01; *: *p* < 0.05; a: significant differences between the less active group and equally active group at baseline; b: significant differences between the less active group and more active group at baseline. n.s.: not significant. Interaction effect: total, F = 57.7, *p* < 0.01; transportation, F = 8.5, *p* < 0.01; light exercise/sports, F = 17.3, *p* < 0.01; moderate or strenuous exercise/sports, F = 14.8, *p* < 0.01; light housework, F = 3.3, *p* < 0.05; moderate or heavy housework, F = 11.2, *p* < 0.01; labor, F = 6.6, *p* < 0.01.

Figure 1 shows the changes in PA following COVID-19 restrictions. According to the results of comparison of PA at baseline among groups, moderate or strenuous exercise/sport in the equally active group was significantly lower than the less active group (mean ± SEM: equally active group, 1.8 ± 0.6 MET h/week; less active group, 5.1 ± 0.8 MET h/week; *p* < 0.05), and resistance exercise/sport in the more active group was significantly lower than the less active group (more active group, 0.8 ± 0.3 MET h/week; less active group, 2.2 ± 0.4 MET h/week; *p* < 0.05). There were no significant differences in other categories of activity at baseline.

In the less active group, a significant decrease was observed as follows: in transportation by 36.6% (baseline, 8.2 ± 1.0 MET h/week; follow-up, 5.2 ± 0.6 MET h/week); light exercise/sports by 65.9% (baseline, 9.1 ± 1.4 MET h/week; follow-up, 3.1 ± 0.6 MET h/week); moderate or strenuous exercise/sports by 80.4% (baseline, 5.1 ± 0.8 MET h/week; follow-up, 1.0 ± 0.3 MET h/week); light housework by 23.5% (baseline, 20.0 ± 2.3 MET h/week; follow-up, 15.3 ± 2.0 MET h/week); and moderate or heavy housework by 28.9% (baseline, 8.3 ± 1.9 MET h/week; follow-up, 5.9 ± 1.5 MET h/week) (*p* < 0.05), which resulted in a significant decrease in total PA by 37.7% (baseline, 60.2 ± 5.9 MET h/week; follow-up, 37.2 ± 4.2 MET h/week; the data were not shown on the figure) (*p* < 0.01).

In contrast, in the more active group a significant increase was observed in light exercise/sports activity by 59.2% (baseline, 4.9 ± 1.5 MET h/week; follow-up, 7.8 ± 1.7 MET h/week); light housework by 20.8% (baseline, 17.8 ± 3.5 MET h/week; follow-up, 21.5 ± 3.3 MET h/week); and moderate or heavy housework by 61.6% (baseline, 11.2 ± 2.9 MET h/week; follow-up, 18.1 ± 4.0 MET h/week) (*p* < 0.05), resulted in a significant increase in total PA by 47.2% (baseline, 52.3 ± 9.1 MET h/week; follow-up, 77.0 ± 11.9 MET h/week; the data are not shown in Figure) (*p* < 0.01).

### 3.4. SWB and HRQoL Scores Based on Changes to Physical Activity

Table 2 summarizes the changes in SWB and HRQoL scores at baseline and follow-up after COVID-19 restrictions. SWB scores significantly decreased in the less active group (mean ± SD: baseline, 14.2 ± 5.1 points; follow-up, 11.7 ± 5.6 points; *p* < 0.01), but this was not seen in the more or equally active group. There were no significant differences in SWB scores across the groups at baseline. No significant interaction effect was observed in the HRQoL score, while the main effect of time was significantly shown in the mental component summary score of HRQoL; HRQoL scores were reduced by COVID-19 restrictions regardless of changes in PA.

### 3.5. Determinants of Decreased Physical Activity

Table 3 represents the results of the univariate and multivariate analyses. In the univariate analysis, the odds of decreased PA were significantly higher in participants who had higher light exercise/sports activity at baseline (odds ratio [OR], 1.04; 95% confidence interval [CI], 1.01–1.07; *p* < 0.01), moderate or strenuous exercise/sports activity at baseline (OR, 1.17; 95% CI, 1.02–1.23; *p* < 0.01), resistance exercise/sports activity at baseline (OR, 1.07; 95% CI, 1.03–1.13; *p* < 0.01), lower mental component score of HRQoL at baseline (OR, 0.97; 95% CI, 0.94–0.99; *p* < 0.05), and hobby activity (OR, 2.04; 95% CI, 1.09–3.08; *p* < 0.05). In multivariate logistic regression analysis, a larger amount of moderate or strenuous exercise/sports activity at baseline (OR, 1.12; 95% CI, 1.01–1.24) and a lower mental component score of HRQoL at baseline (OR, 0.96; 95% CI, 0.93–0.99) were associated with an increased risk of decreased PA.

## 4. Discussion

To our knowledge, this is the first study to determine and assess the risk factors of decreased PA due to the impact of the COVID-19 epidemic and associated public health restrictions on SWB and HRQoL in community-dwelling elderly Japanese. Our data suggest that (1) public health restrictions reduced the PAs of about half of the participants, and decreased PA was associated with lower SWB, (2) those who had more outdoor activities and lower HRQoL before COVID-19 restrictions had an increased risk of decreased PA.

COVID-19 restrictions disrupt the normality of elderly people’s daily lives and enforce social distancing and self-isolation upon them, resulting in about half (47.3%) of the participants being less active and decreasing their PA per week by 37.7% in total. When breaking down by type of PA in the less active group, the light and moderate or strenuous exercise/sports and housework categories were mainly affected. The category of light exercise/sports includes walking for exercise or pleasure and flexibility exercises; moderate or strenuous exercise/sports includes jogging and swimming; and resistance exercise/sports includes using weight machines, etc. [17], which mainly reflects the amount of PA outside the home. During the COVID-19 epidemic, recreational and public facilities such as swimming pools, training gyms, parks, and playgrounds were closed, and local opportunities for health promotion (e.g., exercise classes or karaoke classes) were no longer available. In the less active group, 57.7% of respondents had regular hobbies; however, they might not have had a place for their hobbies after COVID-19 restrictions were enforced. Therefore, to maintain PAs, they had to undertake different activities at different locations. Our results show that the participants might find it difficult to find home activities that are comparable to outdoor ones. Moreover, we found that older adults who were less active were affected not only by outdoor recreational or sports activities but also by transportation for daily needs, including walking or riding a bicycle for errands such as going to a store, which may have contributed to their decreased activity and home confinement. Under such unusual situations, modification of mobility, PA, and habitual exercise routines has been enforced, and barriers to maintaining an active lifestyle are difficult to overcome.

Participants who decreased PA were presumably at home much of the time during the COVID-19 restrictions; however, the housework that they used to engage in before the COVID-19 epidemic was also reduced. Furthermore, we investigated the impact of PA on SWB during the epidemic, and our results show that the reduction of PA was related to a worsening status of SWB. Present results are supported by research currently conducted in Canada and Italy regarding the worsening of psychophysical conditions during a period of forced confinement due to a pandemic, although the targeted age of the subjects differs from our study [3,5]. It has been shown in prospective studies that some individuals increase PA in stressful situations as an effort to cope, while stress has a negative effect on PA among less active individuals experiencing acute stress [20]. The elderly, in particular, show vulnerability to changes in social circumstances, and they are more likely to exhibit avoidance behaviors and psychological distress than others during the 2009 novel influenza (H1N1) epidemic [21]. Research has indicated that autonomous motivation and self-efficacy for PA may play a role in a higher likelihood of engaging in PA [5,22]. The SWB score of elderly individuals who became less active in this study was 11.7 ± 5.6 points: a diagnosis of depression by the Major Depression Inventory (MDI) developed by WHO is recommended if the SWB score of WHO-5-J is less than 13 points [23,24]. Depression, anxiety, and other negative emotions indicated by the decline in SWB may have led to a vicious cycle of further decline in activity, including the housework that they had been regularly doing prior to the COVID-19 epidemic.

In contrast, elderly individuals who became more active increased their PA mainly in housework as well as in the light exercise/sports category, such as walking and flexibility exercises. The category of light housework includes cooking, washing dishes, and vacuuming, whereas moderate or heavy housework involves washing windows, outdoor gardening, yard care, etc. When comparing indoor to outdoor PA, people who exercise outdoors reported decreased feelings of tension, confusion, anger, and depression [25]. Research has indicated that exposure to nature increases positive psychological health outcomes such as happiness, mood, and self-esteem; furthermore, it enhances vitality, and reduces stress [26]. Therefore, older adults who stayed at home were able to spend significantly more time on housework and light exercise/sports; in addition, outdoor activities such as walking and gardening may have contributed to the maintenance of SWB scores. Regular exercise improves one’s self-esteem and sense of well-being [3] and provides fewer depressive and anxiety symptoms; thus, PA intervention may play a protective role in suppressing the stress response for acute mental duress due to COVID-19.

Research has established the benefits of staying active and how home workouts can be instrumental to maintaining activity during the pandemic [27,28]. PA interventions for the elderly need to be safe, efficient, simple, and unsupervised. Aubertin-Leheudre et al. [29] provided examples of unsupervised workouts that included strength, balance, and walking as solutions to care for older adults during the COVID-19 pandemic. Other researchers have suggested that simply increasing the number of daily steps has a positive impact on health status. For example, one way is to take a break from sitting with two-minute walks every 20–30 min at home [30]. Adding a short, outdoor walk or taking the stairs whenever possible are other beneficial measures. During future pandemics, it will be important to propose specific actions similar to these for elderly people who are prone to physical inactivity.

In this study, we determined the risk factors for decreased PA during the COVID-19 epidemic. Our results show that participants who originally enjoyed more moderate or strenuous PA and those with lower HRQoL scores on the mental component summary at baseline were more likely to become less active. As aforementioned, moderate or strenuous exercise and sports are conducted mainly outdoors; therefore, public health measures such as closing recreational and public facilities were one of the reasons for the inability to implement these exercises. Various factors, such as physical and mental health status, age, social status and income, and lifestyle habits such as alcohol consumption, have been reported as reasons for low HRQoL [31,32,33], and it is not possible to clearly reference these factors in this study. However, the most important observation during the epidemic was that those with higher health literacy had a higher HRQoL and were able to maintain PA during COVID-19 [11]. Health literacy is defined as the ability to find, understand, appraise and apply health-related information that could help individuals make appropriate health decisions [34]. Adequate health literacy is a protective factor for improving HRQoL and enables efficient health policy implementation, effective health-promoting efforts, better self-care, and healthcare outcomes [35,36]. Participants with poor HRQoL (considered to have low health literacy) before the COVID-19 epidemic may have failed to integrate much of the information associated with COVID-19 infections, resulting in lower PA and a lower SWB, leading to a secluded life. 

The study has the following strengths. First, we administered a questionnaire to quantitatively measure PA according to intensity and category. Second, the study design allowed us to examine the associations between changes in PA, SWB, and HRQoL outcomes before and during the COVID-19 epidemic, as well as the risk factors associated with reduced PA. However, there are several limitations to this study. First, the measures and changes in PA were self-reported and retrospective, which may have led to recall bias. Second, the current study did not include the elderly with no history of physical illness, which may have led to selection bias and affected the study results. Third, our study sample was very limited compared to other current similar studies with thousands of participants.

## 5. Conclusions

Public health measures reduced the PA of about half of the participants, and PA was strongly associated with SWB. This suggests that psychological and social support that prevents a decline in PA is essential for improving SWB. Those who originally engaged in more outdoor activities are strongly affected by public restrictions and should be provided with alternative opportunities for PA, where feasible. Health promotion measures directed at those with low HRQoL are essential to prevent the worsening of negative behaviors in PA and to maintain community well-being.

To maintain PA and psychological health of the elderly living in the community during a pandemic, the following measures should be considered: (1) help the elderly integrate PA under public health restrictions in a safe, efficient, simple, and unsupervised manner, (2) implement health literacy education to help individuals make appropriate health decisions and acquire better HRQoL, and (3) support elderly individuals who have difficulty increasing their PA, especially those who are psychologically depressed.

## Figures and Tables

**Table 1:** The characteristics of participants.

Characteristics of Participants		Total	Man	Women
		N (%)	N (%)	N (%)
Gender		165 (100.0)	50 (30.3)	115 (69.7)
Age	Mean (years)	78.6 ± 8.0	78.8 ± 8.4	78.6 ± 7.8
	65–69	21 (12.7)	9 (18.0)	12 (10.4)
	70–74	26 (15.8)	6 (12.0)	20 (17.4)
	75–79	43 (26.1)	10 (20.0)	33 (28.7)
	80–84	37 (22.4)	9 (18.0)	28 (24.3)
	85–89	25 (15.2)	11 (22.0)	14 (12.2)
	90 +	13 (7.9)	5 (10.0)	8 (7.0)
Health History	None	24 (14.5)	8 (16.0)	16 (13.9)
	Diabetes	26 (15.8)	9 (18.0)	17 (14.8)
	Hypertension	84 (50.9)	23 (46.0)	61 (53.0)
	Cardiovascular	22 (13.3)	8 (16.0)	14 (12.2)
	Cerebrovascular	28 (17.0)	16 (32.0)	12 (10.4)
	Orthopedic	76 (78.0)	17 (34.0)	59 (51.3)
Employment	Present	9 (5.5)	3 (6.0)	6 (5.2)
	Absent	156 (94.5)	47 (94.0)	109 (94.8)
Hobby	Present	78 (47.3)	25 (50.0)	53 (46.1)
	Absent	87 (52.7)	25 (50.0)	62 (53.9)
Family Structure	Alone	46 (27.9)	6 (12.0)	40 (34.8)
	Elderly couple	72 (43.6)	15 (30.0)	57 (49.6)
	With family	47 (28.5)	9 (58.0)	18 (15.7)
Functional Health	Dependent	96 (58.2)	33 (66.0)	63 (54.8)
	Independent	69 (41.8)	17 (34.0)	52 (45.2)
Residential Density	High	22 (13.3)	5 (10.0)	17 (14.8)
	Low	143 (86.7)	45 (90.0)	98 (85.2)
Access to shops	Good	80 (48.5)	24 (48.0)	56 (48.7)
	Poor	85 (51.5)	26 (52.0)	59 (51.3)
Sidewalks	Present	101 (61.2)	31 (62.0)	70 (60.9)
	Absent	64 (38.8)	19 (38.0)	45 (39.1)

**Table 2:** Subjective well-being (SWB) and health-related quality of life (HRQoL) outcomes in relation to changes in physical activity (PA) since COVID-19.

Variables	Changes in PA Since COVID-19			Interaction Effect (Group × Time)		Main Effect (Time)	
	More Active (*n* = 38)	Equally Active (*n* = 49)	Less Active (*n* = 78)	F Value	*p*-Value	F Value	*p*-Value
SWB							
Baseline	14.8 ± 6.1	13.5 ± 5.4	14.2 ± 5.1	6.98	<0.01	24.9	<0.01
Follow-up	14.6 ± 5.3	13.2 ± 5.6	11.7 ± 5.6 ^a^				
HRQoL							
PCS							
Baseline	38.6 ± 12.8	35.5 ± 12.5	36.9 ± 11.9	0.30	0.74	0.01	0.99
Follow-up	39.0 ± 12.1	34.9 ± 13.2	37.0 ± 11.8				
MCS							
Baseline	53.0 ± 9.6	52.4 ± 9.3	50.1 ± 10.6	0.34	0.71	17.4	<0.01
Follow-up	49.0 ± 10.8	49.8 ± 9.9	47.5 ± 10.7				

SWB, subjective well-being, score of WHP-5-J; HRQoL, health-related quality of life, score of SF-12; PCS, physical component summary; MCS, mental component summary; PA, physical activity. Data are expressed as the mean ± SD (points). ^a^: Significant differences between baseline and follow-up in the less active group (*p* < 0.01).

**Table 3:** Risk Factors Associated with Decreased PA.

Variables	Changes in PA Since COVID-19		Univariate Analysis		Multivariate Analysis	
	More/Equally Active (*n* = 87)	Less Active (*n* = 78)	OR (95%CI)	*p*-Value	OR (95%CI)	*p*-Value
PA at Baseline						
Transportation	5.9 ± 8.3	8.1 ± 9.0	1.03 (0.99, 1.07)	0.10		
Light exercise/sports	4.6 ± 10.4	9.1 ± 12.4	1.04 (1.01, 1.07)	<0.01	1.02 (0.99, 1.06)	0.18
Moderate or strenuous exercise/sports	2.4 ± 4.9	5.1 ± 7.2	1.17 (1.02, 1.23)	<0.01	1.12 (1.01, 1.24)	0.04
Resistance exercise/sports	1.1 ± 2.4	2.2 ± 3.3	1.07 (1.03, 1.13)	< 0.01	1.05 (0.99, 1.10)	0.30
Light housework	16.0 ± 20.3	20.0 ± 20.2	1.01 (0.99, 1.02)	0.22		
Moderate or heavy housework	10.7 ± 19.1	8.3 ± 16.3	0.99 (0.97, 1.01)	0.37		
Labor	8.4 ± 19.4	7.3 ± 19.2	1.00 (0.98, 1.01)	0.67		
SWB at Baseline	14.7 ± 5.6	14.2 ± 5.1	0.98 (0.93, 1.04)	0.55		
HRQoL at Baseline						
PCS	36.8 ± 12.6	36.9 ± 11.9	0.98 (0.92, 1.05)	0.57		
MCS	52.7 ± 10.4	50.1 ± 10.6	0.97 (0.94, 0.99)	0.04	0.96 (0.93, 0.99)	0.03
Gender (for woman)	61 (70.1)	54 (69.2)	0.96 (0.49, 1.90)	0.91		
Age	78.9 ± 7.3	78.3 ± 8.8	0.99 (0.95, 1.02)	0.46		
Health History						
Diabetes	13 (14.9)	13 (16.7)	1.14 (0.49, 2.64)	0.76		
Hypertension	42 (48.3)	45 (57.7)	1.40 (0.75, 2.59)	0.29		
Cardiovascular	11 (12.6)	11 (14.1)	1.14 (0.46, 2.79)	0.78		
Cerebrovascular	18 (20.9)	10 (13.0)	0.56 (0.24, 1.31)	0.12		
Orthopedic	39 (45.3)	37 (48.1)	1.11 (0.60, 2.07)	0.73		
Employment (for presence)	4 (4.6)	5 (6.4)	1.42 (0.37, 5.50)	0.61		
Hobby (for presence)	34 (39.1)	45 (57.7)	2.04 (1.09, 3.08)	0.02	1.63 (0.81, 3.30)	0.17
Family Structure						
Alone	23 (26.4)	23 (29.5)	1.10 (0.55, 2.18)	0.79		
Elderly couple	35 (40.2)	37 (47.4)	1.35 (0.73, 2.52)	0.35		
With family	29 (33.3)	18 (23.1)	1.00			
Functional Health (for independent)	33 (37.9)	36 (46.2)	1.41 (0.76, 2.63)	0.28		
Residential Density (for high)	9 (10.3)	13 (16.7)	1.58 (0.63, 3.98)	0.33		
Access to Shops (for good)	44 (50.6)	36 (46.2)	0.84 (0.45, 1.55)	0.57		
Sidewalks (for presence)	50 (57.5)	51 (65.4)	1.40 (0.74, 2.63)	0.30		

SWB, subjective well-being, score of WHP-5-J; HRQoL, health-related quality of life, score of SF-12; PCS, physical component summary; MCS, mental component summary; PA, physical activity; OR, odds ratio; CI, confidence interval.
